# Promoting Vaccinations in Pregnancy: Results of a Systematic Literature Review of Italian Initiatives

**DOI:** 10.3390/vaccines12030235

**Published:** 2024-02-24

**Authors:** Sara Properzi, Maria Stella Sepioni, Roberta Carestia, Giulia Cervelli, Chiara de Waure

**Affiliations:** 1 Department of Medicine and Surgery, University of Perugia, 06132 Perugia, Italy; roberta.carestia@studenti.unipg.it (R.C.); giulia.cervelli@studenti.unipg.it (G.C.); chiara.dewaure@unipg.it (C.d.W.); 2 Department of Medicine and Surgery, Degree Course in Obstetrics, University of Perugia, 06132 Perugia, Italy; mariastellasep@libero.it

**Keywords:** vaccines, hesitancy, intervention, pregnancy, knowledge, attitudes, behaviors

## Abstract

Pregnant women and infants inherently face heightened susceptibility to complications resulting from infectious diseases. Within these populations, vaccinations offer numerous advantages. This systematic review endeavors to comprehensively analyze the existing literature concerning interventions designed to promote vaccinations among pregnant women and newborns in Italy. We searched PubMed, Scopus, and Web of Science for primary studies published until 3 August 2023 which assessed the impact of vaccination education interventions targeting pregnant Italian women. Data extraction, pooling, and a quality appraisal of the included studies were conducted according to PRISMA guidelines. Among the 528 articles identified, 3 met the inclusion criteria and focused on pregnant women aged 25 to 40 attending pre-delivery courses. In these studies, the effectiveness of the interventions was assessed using pre- and post-intervention questionnaires that investigated knowledge, attitudes, and behaviors regarding recommended vaccinations. The results reveal significant increases in intention and adherence to vaccination among participants after these interventions. The results underscore the positive influence of health professionals’ educational initiatives on pregnant Italian women’s vaccination knowledge and attitudes. However, longitudinal studies with larger representative samples are needed to validate these findings and identify potential avenues for improving maternal educational interventions.

## 1. Introduction

Pregnant women and infants are commonly at an increased risk of complications in the case of infection with a pathogen [1]. Scientific evidence confirms that during pregnancy, there is a modulation of the immune response related to hormonal changes so as to ensure the immunologic tolerance of the semi-allogeneic fetus until the time of delivery [2]. These changes also increase susceptibility to some infections, such as the influenza virus [3,4,5]. Increased susceptibility to some infectious diseases is indeed present, although pregnant women do not completely lack defenses against pathogens [6].

Immunological changes also place pregnant women and fetuses at a greater risk of facing more severe outcomes in the case of infection [7,8,9]. Because of their immature immune systems, newborns and infants also have higher morbidity and mortality rates due to infections [1]. Maternal antibodies passively transferred through the placenta during gestation provide protection to infants in the first months of life [10]. The absence of specific antibodies transmitted by the mother makes the infant vulnerable, at least until the infant reaches the vaccination age and completes the vaccination cycle. Therefore, immunization during pregnancy offers several advantages, namely direct protection for the pregnant woman, a reduction in the likelihood of the maternal–fetal transmission of infection, and passive immunity for the newborn due to the trans-placental transmission of antibodies [11], helping to reduce morbidity and mortality in children under five years of age [12]. Hence, in conjunction with efforts to support the fundamental role of vaccination throughout life, with annual initiatives such as the World Health Organization-supported “World Immunization Week” [13], it is imperative to implement campaigns supporting vaccination during the gestational period, emphasizing the safety and efficacy of vaccines, even in the context of this particular life stage. Indeed, despite the known benefits associated with maternal vaccination and the continuous monitoring of safety through pharmacovigilance systems [14,15,16], concern about vaccine safety and possible side effects in the gestational period remain a common barrier to vaccination among pregnant women [17], resulting in low levels of vaccination coverage during pregnancy worldwide [18,19]. 

Several systematic reviews have addressed the factors influencing vaccine uptake during pregnancy and have called attention to the importance of health promotion initiatives to increase pregnant women’s knowledge, modify their attitudes, and, ultimately, positively impact their vaccination behaviors [20,21,22,23,24,25]. 

To the best of our knowledge, the currently available systematic reviews on strategies to improve vaccination during pregnancy have considered a wide range of interventions that were not focused on pregnant women only. Indeed, in conjunction with patient vaccination education [26,27,28,29], staff education and training [26,27,29], reminder and recall systems for vaccinations [26,27], and interventions involving facilitated vaccine administration by trained staff [26] have demonstrated effectiveness in enhancing knowledge and promoting vaccination adherence among pregnant women. Multidisciplinary interventions, characterized by the simultaneous integration of strategies at the patient, provider, and practice levels, consistently exhibit superior efficacy in fostering adherence to vaccination protocols. Furthermore, the currently available evidence has addressed specific vaccinations [26,27,28,29]. 

With this systematic review, we would like to scrutinize the effect of pregnant-women-focused interventions that have been carried out in Italy on improving knowledge, attitudes, and behaviors toward vaccination.

## 2. Materials and Methods

We conducted a systematic review of the scientific literature according to PRISMA guidelines [30], using three databases (MEDLINE PubMed, Scopus, and Web of Science). The search was conducted until 3 August 2023 and did not have time or language restrictions. Search terms related to pregnancy, vaccination and immunization, and knowledge, attitudes, and behaviors regarding vaccination were included. The full search strategy is shown in Table 1.

### 2.1. Eligibility Criteria

Studies that met the following eligibility criteria, described as PICOS [30], were included: (1) population (P): Italian pregnant women during any trimester of pregnancy; (2) intervention (I): any intervention targeted to pregnant women and aimed at modifying their knowledge, attitudes, and behaviors with respect to vaccination; (3) control (C): no intervention; (4) outcome (O): women’s knowledge/attitudes/behaviors toward vaccinations; and (5) studies (S): studies adopting an experimental or quasi-experimental study design in order to assess the efficacy/effectiveness of the intervention. Observational studies and qualitative investigations were excluded from consideration. 

### 2.2. Study Selection

The first author (SP) imported the literature acquired from searches conducted in three databases into the Rayyan online platform dedicated to systematic reviews [31]. Subsequently, duplicates were removed, and screening based on title and abstract was conducted by two independent reviewers (MSS and GC) in accordance with the abovementioned predefined eligibility criteria. Selected articles were reviewed independently through a full-text analysis for eligibility by two reviewers (SP and RC). Discrepancies and differences between the two reviewers (SP and RC) regarding the selection and inclusion of a particular source were discussed, involving the authors MSS and GC as well, until a consensus was reached. 

**Figure 1:**
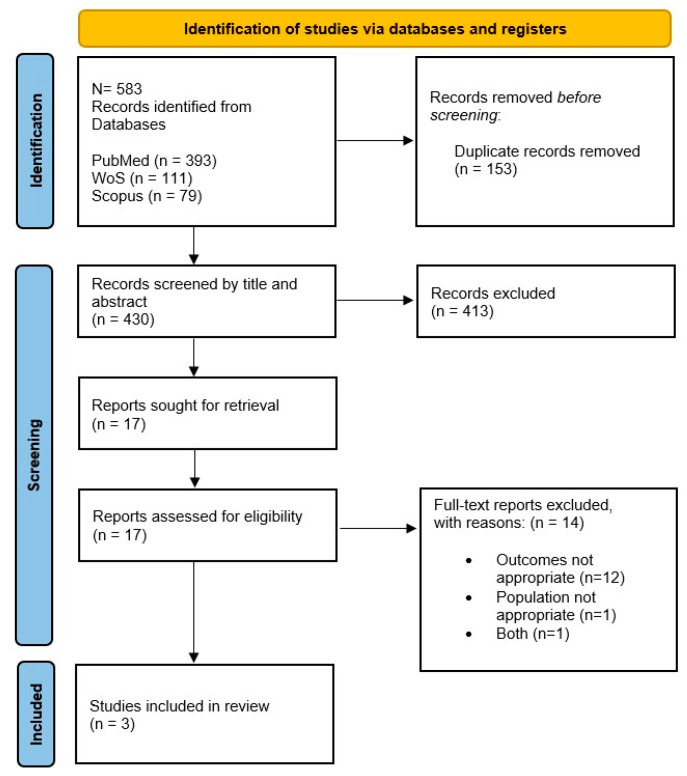
PRISMA flow chart.

Articles excluded at this stage due to an inappropriate population, intervention, and/or outcome were formally recorded as reported in Figure 1. 

### 2.3. Data Extraction

Data from the included publications were extracted and entered into a standard data extraction form. We extracted the following data: the first author’s name, year of publication, study design, study location, study period, study population, sample size, sample age, type of intervention, intervention characteristics (type, duration, and structure), endpoints and tools for endpoints assessments, and main results.

### 2.4. Data Synthesis

In the context of this systematic review, a qualitative synthesis of the data was performed according to a thematic synthesis approach with respect to an a priori set of themes, namely pregnant women’s knowledge, attitudes, and behaviors. This approach was adopted as even though the primary studies yielded quantitative results, they were not homogenous enough to be combined through a meta-analysis. Data referring to the pre- and post-intervention knowledge, attitudes, and behaviors of pregnant women from each study were reported and evaluated in terms of significant differences. Results are described in separate paragraphs in order to make it easier to compare the findings of included studies. 

### 2.5. Methodological Quality (Risk of Bias) Assessment 

For a quality assessment, the JBI critical appraisal checklist for an analytical cross-sectional study [32] was employed by examining the following 8 aspects: the precision of the inclusion criteria, transparency regarding the subject and study setting, clarity in the methodology employed for exposure measurement, the utilization of objective and standardized criteria for condition measurements, the identification of confounding factors, the management of confounding factors, the establishment of a valid and reliable outcome measurement, and the execution of appropriate statistical analyses. A score of 1 was assigned for each evaluated aspect in case of an affirmative response.

The included papers were evaluated by two independent reviewers (SP and RC). Disagreements were resolved through a discussion among the reviewers and, in the second instance, with the involvement of the third reviewer (GC).

## 3. Results

### 3.1. Study Selection

The initial search of the three electronic databases yielded 583 potential studies. After the removal of duplicates (153) and exclusions on the basis of title and abstract (413), the full texts of 17 studies were evaluated for possible inclusion. Fourteen studies were excluded for the following reasons: inappropriate outcomes (12), different study population (1), and both (1).

Three papers [33,34,35] which met all inclusion criteria were considered in the review (Figure 1).

### 3.2. Study Characteristics

All three studies included in this review adopted a repeated cross-sectional study design, consistent with a quasi-experimental (pre–post) study design. The overall quality of the papers was rated as moderate–high, with scores ranging from 6/8 [33] to 7/8 [34,35]. The studies had limitations stemming from potentially inadequate representation due to the restricted number of participants and the lack of control over the enrolment process by the researchers [34,35]. Furthermore, a considerable selection bias may be represented by the fact that the women involved in the preparatory courses had a higher level of education than the general population [34,35]. It should also be considered that the gestational age at the time of the compilation of the pre- and post-intervention questionnaires was not documented, precluding an assessment of potential influences on the answers given in relation to the stage of pregnancy [33]. The three studies were conducted within hospital facilities in the metropolitan areas of Florence [33], Rome [34], and Palermo [35] and involved pregnant women in different trimesters of pregnancy, with the number of participants ranging from 119 to 326 (Table 2). The study populations consisted of people participating in childbirth preparation classes [33,34,35]. Notably, one of the studies also extended invitations to women engaged in prenatal diagnostic consultations for congenital anomalies [33]. 

### 3.3. Intervention Characteristics

The interventions were been implemented during childbirth preparatory courses that were held with varying frequency in attendance [33,34,35]. Since April 2020, due to the COVID-19 pandemic, one of the preparatory courses has been delivered online through digital platforms [35].

The interventions, characterized by 30–60 min sessions led by highly qualified HCWs, mainly physicians, provided information concerning the importance of immunization for childbearing, pregnancy, and the unborn child, with a focus on vaccines, their functions, composition, and adverse effects and false myths. 

A key component of the intervention was the presentation of the vaccination calendar recommended by the NIP in line with its epidemiological and biological rationale. In the study conducted in Palermo, participants were given the opportunity to request additional vaccine counseling to address lingering doubts or concerns [35]. 

To assess the effect of the interventions, the two studies conducted in Florence and in Rome used a questionnaire adapted from a validated tool [36], obtaining in one case a response rate of 95.7% in both the pre- and post-intervention periods [33], while the other achieved response rates of 87.4% and 66.4% in the pre- and post-intervention periods, respectively [34]. 

In one study [35], the pre-intervention survey, to which 100% of the enrolled women responded, was performed through a questionnaire validated in a preliminary pilot study. 

The questionnaire contained 36 items aimed at collecting socio-demographic data and vaccination knowledge and attitudes. The post-intervention assessment was performed after 30 days by text message and/or WhatsApp message or e-mail contact to assess adherence to flu vaccination and/or diphtheria–tetanus–pertussis acellularis (DTPa) and the main reason for refusing vaccination, with 62% of participants participating.

The data showed that the main sources of information regarding vaccination were word of mouth (friends, family members, etc.) (50%) [33], health professionals, particularly family doctors (27.9–45.7%) [33,35], traditional mass media (TV, radio, and newspapers) (35.7%) [33], and institutional websites (19.5–24.2%) [34,35]. Specialists such as pediatricians and gynecologists were consulted less frequently (16.2–21.4%) [33].

### 3.4. Effects on Knowledge

Pregnant women’s knowledge was assessed in all three included studies. 

The participants’ knowledge of the danger of infectious diseases, both for the pregnant woman and the unborn child, was found to be low. Indeed, in the pre-intervention survey in the study conducted in Rome, 40.5% recognized the danger of C. diphtheriae infection and only 35.6% recognized that of H. influenzae infection [34], pathogens responsible for serious complications in pregnant women and unborn children [37,38,39]. Following the intervention, there was a significant surge in the proportion of individuals recognizing the danger of C. diphtheriae infection, escalating to 61.8%. Additionally, a rise to 54.1%, although not significant, was also observed in the proportion of individuals acknowledging the peril of H. influenzae infection [34]. 

The investigation undertaken at Palermo Hospital [35] during the pre-intervention phase assessed the extent of awareness concerning potential risks posed by infectious diseases for the mother, fetus, and newborn during the initial months of life; slightly more than half of the participants, precisely 57.1%, demonstrated an awareness of the potential impact of severe complications associated with influenza, and only 36.5% were aware of the possible complications resulting from B. pertussis infection [35]. In the same study [35], it emerged that while 70% of the interviewees were aware of the recommendation for influenza vaccination during pregnancy, merely 32.8% of pregnant women were informed about the necessity of receiving a DTPa vaccine booster in each pregnancy.

During the pre-intervention period, 15% of participants in the study conducted in Florence [33] reported direct or indirect personal experiences of one or more post-vaccination side effects encompassing severe conditions such as autism, meningitis, deafness, polio, and acute leukemia. However, subsequent to the intervention, this percentage exhibited a decline, suggesting that the instances reported in the pre-intervention survey were likely influenced by unfounded beliefs or misinformation rather than authentic personal experiences. Therefore, regarding one of the most prevalent items of vaccine-related misinformation, the purported causal relationship between vaccines and autism, findings from the two studies conducted in Florence and Rome [33,34] indicated a significant increase in the proportion of individuals who did not recognize the existence of this causal relationship after the intervention, increasing from 43.8% [33] and 41% [34] in the pre-intervention period to 84% [33] and 73% [34] in the post-intervention period. 

In the study conducted in Florence, in evaluating general knowledge levels in the domain of vaccines, there was a notable decrease from 43% to 13% in responses signifying a low level of knowledge after the intervention [33]. Furthermore, in the post-intervention period, 64.6% of participants in the Rome study [34] found the session a useful tool for obtaining information compared with 30.3% in the pre-intervention period (*p* < 0.001).

Overall, in the examined studies, pregnant women initially displayed a limited level of understanding of the risks associated with infectious diseases for themselves and their offspring [33,35]; however, the interventions in the three studies consistently reported a marked improvement in knowledge regardless of the instruments used to assess it [33,34,35].

### 3.5. Effects on Attitudes

Two out of the three studies issued results on pregnant women’s attitudes, reporting an overall increase in the women’s intention to vaccinate during pregnancy and to also subject their offspring to vaccination after the intervention [33,34].

The study conducted in Florence showed that prior to the intervention, the average score of items indicating the propensity to undergo vaccination during pregnancy and to administer vaccinations to offspring was 35.5 (95% CI: 33.6–37.3), and it increased significantly to 42.6 (95% CI: 41.3–43.8) after the intervention [33]. The scoring procedure involved assigning a value of “0” to responses expressing an opposition to vaccination, a value of “1” to responses characterized as neutral or hesitant, and a value of “3” to responses endorsing vaccination [33].

The results of both studies addressing pregnant women’s attitudes [33,34] agreed that there was a substantial increase in the intention to undergo several vaccinations after the intervention (Table 3). 

After the intervention, a significant increase in the inclination toward vaccination against H. influenzae was observed in the participants of the Florence study [33]. Specifically, 87% demonstrated a predisposition to undergo vaccination for both themselves and their offspring compared to pre-intervention rates of 52% [33] (Table 3). A notable, albeit not significant, augmentation in the propensity to undergo vaccination for both the participants and their unborn children against H. influenzae was likewise observed among the participants in the Rome study. This inclination rose from 58.7% in the pre-intervention period to 70.9% in the post-intervention period [34].

Moreover, among the participants, there was a significant increase in preference expressed for tetanus vaccination. Notably, the proportions rose from 78% in the Florence study and 80.8% in the Rome study before the intervention to 92% and 91.1%, respectively, after the intervention [33,34].

Furthermore, among participants in the study carried out in Rome [34], a significant surge in preference for Human Papilloma Virus (HPV) vaccinations was discerned after the intervention. Specifically, the preference increased from 51% to 65.8% for HPV vaccination when comparing the pre-intervention phase to the post-intervention phase [34]. 

The percentage of those who considered the vaccination calendar an effective means of attaining direct and indirect protection for their offspring significantly increased by 18.18% and 12.52%, respectively, after the intervention [34].

The intervention had no significant effect on the opinion on mandatory vaccination, with 98.7% in favor after the intervention compared to 96.04% in the pre-intervention period in the study conducted in Rome [34] and 10% against it after the intervention compared to 11% in the pre-intervention period in the study conducted in Florence [33].Overall, the intervention led to a significant increase in the intention to undergo various vaccinations for both the women and their offspring [33,34]. Additionally, the intervention positively influenced perceptions regarding the importance of the vaccination schedule for offspring protection, while it did not bring about any significant changes in opinions regarding compulsory vaccination [33,34].

### 3.6. Effects on Behavior

Behaviors toward vaccinations were addressed in two out of the three studies [34,35]. Women participating in the study carried out in Rome and their carers were offered a free influenza vaccination at the end of the intervention or on other agreed-upon dates [34]. Following the educational intervention, during the study, 48 (40.34%) women out of the 119 participants in the antenatal course were vaccinated against influenza, 46 of them onsite and 2 at the General Obstetric Clinic [34]. Furthermore, 39 of their partners (32.8%) also received the influenza vaccination post intervention [34]. Throughout the observational period at the established vaccination facility, a cohort comprising five female individuals who did not engage in the educational intervention also received vaccinations against influenza [34].

At least 30 days after the intervention, 62% of participants in the research study conducted in Palermo submitted feedback through either email or text message. Among them, 47.8% (+44.8% compared to the pre-intervention period) stated that they had received the influenza vaccination during the current pregnancy, 57.7% (+50.7% compared to the pre-intervention period) stated that they had received the DTPa vaccination, and 64.2% (+54.8% compared to the pre-intervention) stated that they had received at least one of the recommended vaccinations, showing a significant increase in vaccination adherence post intervention [35]. The remaining 38% did not furnish information regarding whether or not they had undergone the offered influenza and anti-DTPa vaccinations [35].

There was also a significant association between adherence to at least one recommended vaccination and a higher level of education (graduate degree/Master’s degree) compared to a lower level of education (high school/primary–secondary school diploma) (adjusted OR = 3.12; 95% CI 1.25–4.67), employment (part-time/full-time) compared to unemployment (adjusted OR = 1.89; 95% CI 1.11–5.23), and women who received at least one vaccination during the last five influenza seasons compared to those who had received none (adjusted OR = 4.12; 95% CI 2.06–5.46) [35].

Despite the implementation of the intervention in the study conducted in Palermo, it was observed that 47.6% of vaccine refusals 30 days after the intervention still originated from women expressing apprehension about potential adverse effects [35]. Furthermore, it was documented that 43% of post-intervention vaccination refusals still stemmed from women who had not received a vaccination recommendation from their obstetrician/gynecologist [35]. In conclusion, despite the significant increase in vaccination uptake after the intervention, there is still room for improvement, and further evidence is needed to identify the best strategies for reaching the entire target population.

## 4. Discussion

The primary aim of this study was to collate evidence on the impact of pregnant-women-focused interventions on the knowledge, attitudes, and behaviors of pregnant women toward vaccination in the Italian context. The three studies included foresaw the implementation of an educational intervention held during childbirth preparation courses and were therefore comparable in terms of settings and type of intervention. Unfortunately, comparability was lower regarding the outcome assessment because of the diversity of considered endpoints and adopted instruments. Nevertheless, in our opinion, a positive conclusion can be drawn. 

### 4.1. Summary of Findings

Prior to the intervention, a significant proportion of women exhibited limited awareness of the potential risks associated with vaccine-preventable infectious diseases. Following the intervention, a substantial improvement in the recognition of these risks was observed across the studies, indicating the effectiveness of the intervention. This result is important in light of enhancing the participants’ understanding of the importance of vaccination during pregnancy, both for their own well-being and the health of their newborns.

In fact, the lack of knowledge regarding the impact of vaccine-preventable diseases, and with respect to vaccine effectiveness, the persistence of misconceptions about vaccination side effects remain among the primary reasons for vaccine refusal [35,40].

Consistent with previous studies [41,42], our empirical findings substantiate the pivotal role of HCWs in influencing patients’ vaccination decisions. Therefore, it is essential to not only enhance interventions aimed at reaching the vaccination target population but to also provide ongoing vaccination training for HCWs in line with national recommendations.

The data revealed that interventions, beyond successfully improving knowledge regarding vaccinations, positively contributed to an increase in the intention to receive vaccinations during pregnancy, with a significant rise in adherence to recommended vaccinations [33,34]. The recent literature also indicates that educational interventions administered by HCWs specialized in vaccine-related education prove more efficacious in shaping the knowledge, attitudes, and behaviors of pregnant women regarding vaccines in comparison to digital interventions, such as the dispatch of text messages to pregnant women for reminders, encouragement, or informational purposes pertaining to vaccinations during pregnancy [43,44,45].

It is noteworthy to mention a significant association between adherence to at least one recommended vaccination and higher education levels [35,46,47]. This suggests the need to tailor educational interventions to the educational and socio-demographic background of the target population as these factors can influence vaccination decisions. In this respect, our work offers new relevant insights compared to the systematic reviews available, which mostly focused on the uptake of vaccination, demonstrating that educational interventions were effective in increasing the uptake of both pertussis and influenza vaccinations [26,27,28,29]. Moreover, our review also addressed the role of this type of intervention in modifying knowledge and attitudes that influence the final decision to vaccinate. Indeed, by simultaneously considering knowledge, attitudes, and vaccination adherence, our work provides a broader assessment of the effectiveness of educational interventions.

On the other hand, it is interesting to note that the intervention did not significantly alter participants’ opinions regarding mandatory vaccinations, suggesting that maternal and child vaccination attention might not influence broader beliefs about vaccinations [33]. Consequently, public health policies and communication strategies may need to address these issues separately.

### 4.2. Limitations and Implications for Future Research

Is essential to acknowledge certain limitations of this study. 

A notable constraint in this systematic review stemmed from the limited number of included studies, despite the sensitive approach taken in conducting the research and in avoiding selection bias. 

Furthermore, all three studies included [33,34,35] relied on convenience samples which might not be fully representative of the broader pregnant population. 

To address the identified constraints and improve the generalizability of the findings, future studies may consider expanding the inclusion of different geographic areas and considering other settings. Employing more representative sampling methods, in addition to convenience samples, would contribute to a more complete understanding of the impact of such an intervention. 

We must also consider the potential selection bias within the included studies that may arise from the fact that pregnant women attending childbirth preparation courses might tend to have higher educational levels than the general population [35]. It would be useful for subsequent research to explore the effectiveness of similar interventions in populations with different educational backgrounds, addressing the potential selection bias identified in studies based on convenience samples. Conducting studies in different contexts would provide a more nuanced understanding of the broader impact of such interventions. Additionally, the three studies [33,34,35] were conducted in specific Italian metropolitan areas, limiting the generalizability of the results to other geographical regions and populations. Anonymity during data collection prevented data pairing which, combined with the reduction in the number of post-intervention responses, may introduce a bias. Future investigations could delve deeper into developing strategies to mitigate all these potential biases and concerns. 

Eventually, although the systematic review strictly followed PRISMA guidelines, a selection bias could not be completely ruled out. Furthermore, unfortunately, we could not provide any quantitative summary because of the different outcome measures and tools used in the included studies.

In the future, it may be beneficial to standardize the content of educational interventions and their subsequent evaluations and to focus on assessing their impact in terms of maternal and childhood vaccination uptake as well. Comparative studies conducted across different settings and relying on different tools, including digital ones, could detect best practices that can be further implemented. 

## 5. Conclusions

Immunization through vaccination represents a crucial element in preventing severe diseases and safeguarding individuals’ health and well-being, particularly during phases of life marked by heightened vulnerability or further risk, such as pregnancy. The findings of this systematic review of studies assessing the effect of interventions tailored to pregnant women show that they can increase knowledge and positively influence attitudes and behaviors. Therefore, it would be desirable for educational interventions in the realm of vaccination, conducted by adequately trained HCWs, to become a standard practice, possibly extended to regional and national levels.

## Figures and Tables

**Table 1:** Search strategy.

Search Engine	Search Strategy
MEDLINE/ PubMed	From 1 January 1000 to 3 August 2023: (strategy OR intervention OR program) AND (vaccination OR immunization) AND (pregnancy OR pregnant OR antenatal OR ante-partum) AND (knowledge OR attitudes OR behaviour OR belief OR coverage OR uptake OR trust OR mistrust OR perception OR hesitancy OR confidence OR acceptance OR adherence) AND (Italy OR Italian)
Web of Science	From 1 January 1900 to 3 August 2023: Query: TS = ((strategy OR intervention OR program) AND (vaccination OR immunization) AND (pregnancy OR pregnant OR antenatal OR ante-partum) AND (knowledge OR attitudes OR behaviour OR belief OR coverage OR uptake OR trust OR mistrust OR perception OR hesitancy OR confidence OR acceptance OR adherence) AND (Italy OR Italian))
Scopus	At 3 August 2023: (TITLE-ABS-KEY ((strategy OR intervention OR program)) AND TITLE-ABS-KEY ((vaccination OR immunization)) AND TITLE-ABS-KEY (pregnancy OR pregnant OR antenatal OR ante-partum)) AND TITLE-ABS-KEY ((knowledge OR attitudes OR behaviour OR belief OR coverage OR uptake OR trust OR mistrust OR perception OR hesitancy OR confidence OR acceptance OR adherence)) AND TITLE-ABS-KEY ((italy OR italian))

**Table 2:** Characteristics of studies.

Author and Year	Study Design	Period	City and Setting	Study Population	Sample Size	Participants’ Age
Bechini et al., 2019 [33]	Before–after cross- sectional study	From October 2017 to May 2018	Florence/ Hospital	Pregnant women attending childbirth preparation courses a/o prenatal diagnostic counseling on congenital defects	210	Mean: 34
S. Bruno et al., 2021 [34]	Before–after cross- sectional study	From October 2019 to January 2020	Rome/ Hospital	Pregnant women attending childbirth preparation courses	119	Mean (+SD): 34.5 (+4.9) before intervention; 34.8 (+5.1) post intervention
Costantino et al., 2021 [35]	Before–after cross- sectional study	From October 2019 to October 2020	Palermo/ Hospital	Pregnant women attending childbirth preparation courses	326	>18

**Table 3:** Pre- and post-intervention answers concerning the intention to receive the recommended vaccinations during pregnancy and to vaccinate the future neonate.

Author and Year	Pre N (%)			Post N (%)			Pre–Post (%)		
	Diphtheria	Hib	Poliomyelitis	Diphtheria	Hib	Poliomyelitis	Diphtheria	Hib	Poliomyelitis
S. Bruno et al., 2021 [34]	81/104 (77.9)	61/104 (58.7)	79/104 (76)	69/79 (87.3)	56/79 (70.9)	67/79 (84.8)	+9.4	+12.2	+8.8
A. Bechini et al., 2019 [33]	139/210 (66)	110/210 (52)	152/210 (72)	177/201 (88)	174/201 (87)	171/201 (85)	+22	+35	+13

## Data Availability

Not applicable.
